# Relationship Between Orthostatic Cardiovascular Responses and Anthropometric Indices in Apparently Healthy Young Female Adults

**DOI:** 10.7759/cureus.81569

**Published:** 2025-04-01

**Authors:** Mayowa Adeniyi, Ayoola Awosika, Ayobami A Adamu

**Affiliations:** 1 Department of Physiology (Environmental Physiology), Federal University of Health Sciences, Otupko, NGA; 2 College of Medicine, University of Illinois, Chicago, USA; 3 Department of Family Medicine, Royal Alexandra Hospital, Edmonton, CAN

**Keywords:** anthropometric indices, diastolic blood pressure, orthostasis, pulse pressure, shock index, systolic blood pressure (sbp)

## Abstract

Background

Understanding the relationship between orthostatic cardiovascular responses and anthropometric indices can provide insight into cardiovascular regulation, autonomic function, and disease risk stratification. Orthostatic responses like changes in systolic blood pressure, diastolic blood pressure, and pulse rate can be potentially influenced by body composition, height, weight, and body mass index (BMI). These anthropometric factors play a crucial role in maintaining hemodynamic stability during postural changes due to their complex interaction in modifying factors like systemic vascular resistance, cardiac output, and autonomic control. Alteration in this systemic interaction can be reflected by a change in the shock index (SI) and double product (DP), serving as surrogate markers for cardiovascular efficiency and myocardial workload, respectively. This study aims to investigate how the orthostatic response and the anthropometric indices relationship can be used to assess an individual's vulnerability to dysautonomia, hypotension, or cardiovascular stress during orthostasis.

Materials and methods

Thirty healthy female individuals, averaging 19.5 years, who satisfied the inclusion criteria were selected for the study. Body weight, height, and body mass index were measured. Blood pressure, pulse rate, and other parameters were determined at baseline (sitting position) and after 10 minutes of standing using standard procedures. Statistical significance was accepted at P<0.05.

Results

Although there was no correlation between the body mass index and cardiovascular parameters after 10 minutes of standing, body weight showed a strong positive correlation with the orthostatic shock index (r=0.803). On the other hand, height correlated negatively with orthostatic systolic blood pressure (r=-0.719) and orthostatic pulse pressure (r=-0.702), respectively.

Conclusion

The study's findings suggest that body weight and height can be used to predict orthostatic shock index, systolic blood pressure, and pulse pressure in young adult females. Assessing these orthostatic responses can help refine cardiovascular risk assessments, contributing to personalized medical interventions and improved long-term health outcomes.

## Introduction

Orthostasis refers to the physiological process of maintaining blood pressure and cerebral perfusion upon assuming an upright posture [1]. As a stressor, sudden orthostasis is known to cause diversion of blood to the lower extremities, reduction in venous return, and stroke volume [2,3]. Under this situation, physiologic compensatory activity involves the inactivation of baroreceptors with attendant sympathetic stimulation, and this leads to an increase in heart rate and diastolic blood pressure [4-6]. Additionally, skeletal muscle contractions aid venous return, counteracting gravitational blood pooling. However, prolonged orthostasis is compensated through the activation of the renin-angiotensin-aldosterone system and increased secretions of antidiuretic hormone and cortisol [1,7]. Despite this, standing for at least 2 hours in a 24-hour day for the maintenance of health has been canvassed [7]. Dysfunction in this mechanism can lead to orthostatic hypotension, dizziness, or syncope, particularly in autonomic disorders.

Besides the hemodynamic changes caused by prolonged standing, prolonged orthostasis at the workplace has been linked to adverse health outcomes such as leg pain and lower back pain [8]. A study involving 2165 workers drawn from various occupations was conducted by Bahk et al. [9]. The prevalence of varicose veins and nocturnal leg cramps was reported to be higher among women than men. Tüchsen et al. reported relative risk ratios of 1.85 and 2.63 for hospitalization due to varicose veins for men and women, respectively, who were occupationally exposed to prolonged orthostasis [10]. Among the other adverse effects of prolonged standing are a high risk of thrombotic events [11,12], tachycardia, fatigue, and dizziness, evidenced by increased alpha wave frequency and high alpha/beta ratio on the electroencephalogram [3].

Strategies devised to improve orthostatic tolerance include the use of compression stockings, ingestion of glucose, standing on mats, ingestion of water, sit-stand alternation, consumption of cold drinks, and pre-orthostatic exercise, among others [2,13-18]. Strategies such as the consumption of cold drinks, pre-orthostasis exercise, ingestion of glucose, normal saline, and water work by improving sympathetic activations and muscle sympathetic nerve activity. The use of mats and sit-stand alternation will reduce the impact of gravitational exertion, while stocking will reduce the compliance of peripheral veins and facilitate venous return.

Anthropometric indices, including weight and body mass index, are important tools for health and wellness in the diagnosis of diseases. They are also predictors of susceptibility to anomalies and diseases. In a previous study, the sitting peak expiratory flow rate was found to correlate negatively with body mass index in sanitary workers [19]. A positive correlation was reported between the insomnia index and the body mass index in age-matched female students who observed regular night study [20]. This study aimed to determine whether there are relationships between orthostatic cardiovascular responses and anthropometric indices in healthy young females.

Research questions

The research questions investigated were: What is the relationship between weight and indices of cardiovascular function in young adult females?; What is the relationship between height and indices of cardiovascular function in young adult females?; What is the relationship between body mass index and indices of cardiovascular function in young adult females?; Does upright standing for 10 minutes affect peripheral oxygen saturation in young adult females?

## Materials and methods

Study design

The study adopted an experimental research design whereby 40 adult females were recruited for the study using respondent-driven sampling. Of these, 30 healthy young females, averaging 19.5 years, satisfied the inclusion criteria and were selected for the study.

Exclusion and inclusion criteria

Written consent was obtained from each participant, and a well-structured questionnaire was administered to rule out those with a medical history of respiratory diseases, cardiovascular, kidney, hepatic, and metabolic diseases or anatomical deformities. History of smoking, alcoholism, caffeine, and any form of medication was also taken, followed by physical examination. Participants with musculoskeletal abnormalities, high blood pressure, and male individuals were excluded. Subjects between ages 16 and 20, female, systolic blood pressure (90-119), diastolic blood pressure (60-80), pulse rate (60-100BPM), and respiratory rate (12-20 cycles/min) were considered for the study.

Experimental protocol

The study was done within one week in the Physiology Laboratory, College of Medicine, Federal University of Health Sciences, Otukpo, Nigeria, at room temperature set to 20 degrees Celsius. Participants came in the morning after overnight fasting. They were tutored on experimental procedures, including how to assume an upright position and correspondence techniques in cases where there was a perception of dizziness and unconsciousness. Before commencing the experiment, participants were asked to relax quietly in a sitting position for 10 minutes. Resting blood pressure, pulse rate, and anthropometrical data, such as weight, height, and body mass index, were measured as mean± standard error of the mean.

Determination of Anthropometrical Indices

Body weight was measured using a weighing scale (Hanson, China) to the nearest 0.5 kg. Height was measured using a meter rule calibrated in inches. The BMI of each subject was calculated using the formula weight in kg/square of metric height.

Ten-Minute Orthostasis

Each participant assumed an upright posture from a sitting position and was unsupported without swaying on a flat surface floor for a period of 10 minutes. The stopwatch was started when the participants stood up and was stopped after 10 minutes in the standing position.

Measurement of Pulse Rate and Blood Pressure

The pulse rate was determined from the radial artery using the palpatory technique. Baseline readings were taken in the sitting position and after 10 minutes orthostasis in the standing position.

Blood pressure was measured from the arm, an inch above the elbow, using an automated Omron BP7000 Evolve Wireless Upper Arm Sphygmomanometer (Iris Global Care, China). Baseline readings were taken in a sitting position, as previously reported [21-24]. Blood pressure measurements were obtained after 10-min orthostasis.

Pulse pressure was determined by subtracting diastolic blood pressure from systolic blood pressure. Mean arterial blood pressure was obtained using diastolic blood pressure +1/3 of pulse pressure. The shock index was determined by dividing pulse rate by systolic blood pressure (pulse rate/systolic blood pressure). Double product was obtained by multiplying mean arterial blood pressure by pulse rate.

Statistical analysis

Statistical analysis was conducted using Statistical Package for Social Science Students (SPSS) 23. A statistical test was done using a student t-test. The statistically significant difference was accepted at P<0.05. Correlation of variables was done using Pearson correlation.

## Results

Anthropometrical characteristics and age of the participants

The weight, height, BMI, and age were estimated (Table 1).

**Table 1:** Physical characteristics of the subjects Kg: kilogram; m: meter; yr: year; SEM: standard error of mean *P<0.05

Parameters	Mean ± SEM
Weight (Kg)	57.7 ± 1.56
Height (m)	1.71 ± .018
Body Mass Index	19.7254 ± 0.539
Age (yr)	19.5 ± 0.129

Effect of 10-min orthostasis on cardiovascular parameters

When compared to the baseline group (Table 2), 10-min orthostasis caused significant increases in pulse rate, systolic blood pressure, diastolic blood pressure, mean arterial blood pressure, shock index, and double product with a percentage change of 19, 2.2, 3.5, 3, 14.3, and 22.4, respectively. 10-min orthostasis had no significant effect on pulse pressure and no change from baseline value.

**Table 2:** Effect of 10-min orthostasis on cardiovascular parameters SEM: standard error of mean; *P<0.05 from the baseline group

Cardiovascular parameters	Baseline (Mean ± SEM)	10-min orthostasis (Mean ± SEM)	Percentage change (%)
Pulse rate	77.3 ± 0.925	92.0 ± 1.653*	19.0
Systolic blood pressure	109.8 ± 0.796	112.3 ± 0.775*	2.2
Diastolic blood pressure	71.8 ± 0.580	74.3 ± 0.551*	3.5
Pulse pressure	38.0 ± 0.599	38.0 ± 0.462	0.0
Mean arterial blood pressure	84.4 ± 0.596	87.0 ± 0.596*	3.0
Shock index	0.7 ± 0.009	0.8 ± 0.018*	14.3
Double product	6535.4 ± 99.919	7999.4 ± 145.311*	22.4

Effect of 10-min orthostasis on peripheral oxygen saturation

**Figure 1:**
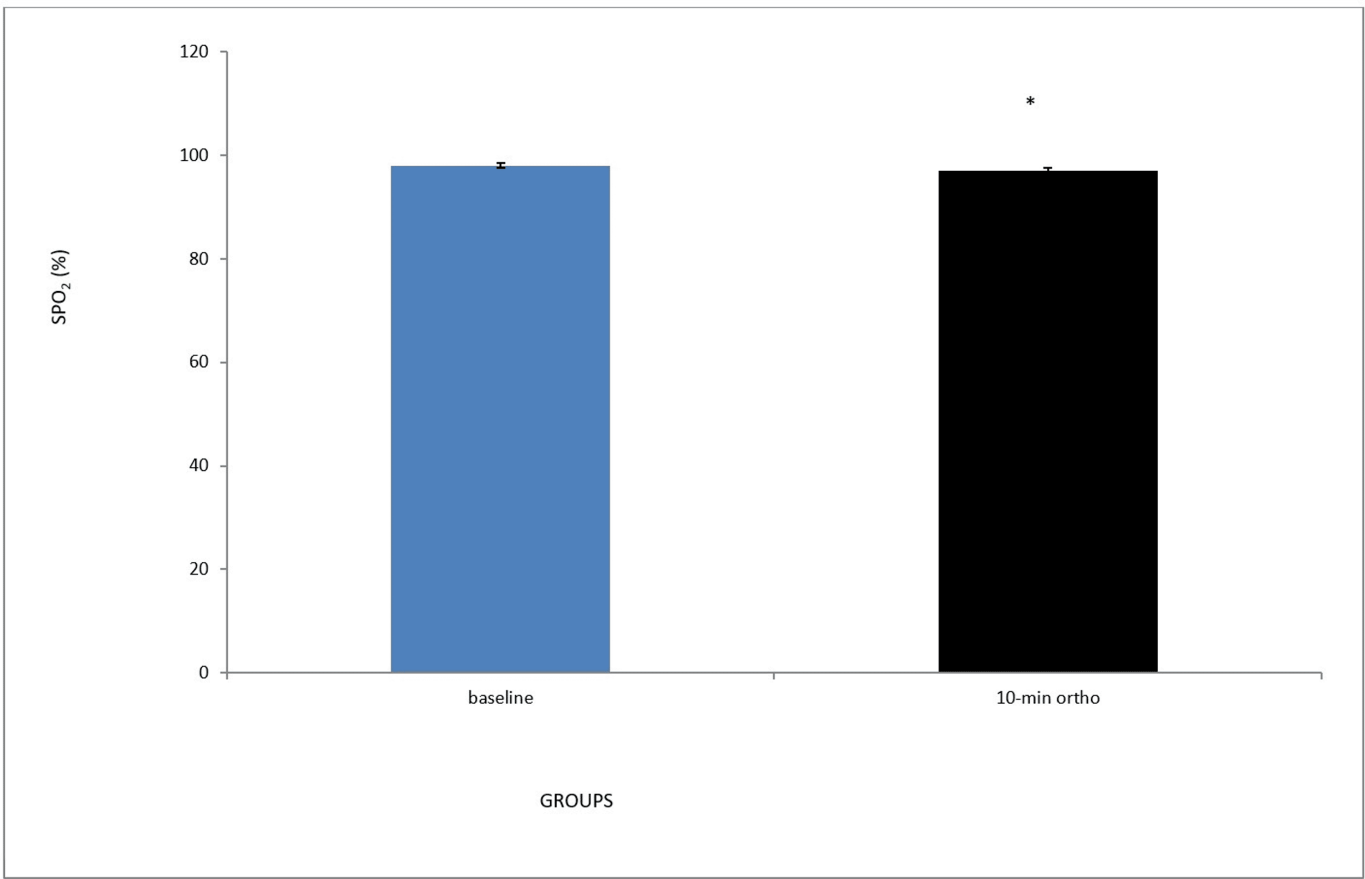
Effect of 10-min orthostasis on peripheral oxygen saturation SpO2: saturation of peripheral oxygen *P<0.05

When compared to the baseline group, peripheral oxygen saturation was significantly reduced during 10-min orthostasis (Figure 1).

Correlation between body weight and orthostatic cardiovascular responses

There is a significant and strong positive correlation between body weight and orthostatic shock index (Table 3). Body weight has no significant correlation with orthostatic systolic blood pressure, orthostatic diastolic blood pressure, orthostatic pulse pressure, orthostatic mean arterial blood pressure, and orthostatic double product.

**Table 3:** Correlation between body weight and orthostatic cardiovascular responses *P< 0.05

Pearson correlation (r)	Body weight
Orthostatic pulse rate	0.53
Orthostatic systolic blood pressure	-0.575
Orthostatic diastolic blood pressure	-0.485
Orthostatic pulse pressure	-0.385
Orthostatic mean arterial blood pressure	-0.548
Orthostatic shock index	0.803*
Orthostatic double product	0.53

Correlation between body height and orthostatic cardiovascular responses

Body height showed a significant strong negative correlation with orthostatic systolic blood pressure and orthostatic pulse pressure (Table 4). Body height has no significant correlation with orthostatic pulse rate, orthostatic diastolic blood pressure, orthostatic shock index, orthostatic mean arterial blood pressure, and orthostatic double product.

**Table 4:** Correlation between height and orthostatic cardiovascular responses *P< 0.05

Pearson correlation (r)	Body height
Orthostatic pulse rate	0.162
Orthostatic systolic blood pressure	-0.719*
Orthostatic diastolic blood pressure	-0.422
Orthostatic pulse pressure	-0.702*
Orthostatic mean arterial blood pressure	-0.571
Orthostatic shock index	0.582
Orthostatic double product	0.24

Correlation between body mass index and orthostatic cardiovascular responses

According to Table 5, body mass index showed no significant strong correlations with orthostatic pulse rate, orthostatic systolic blood pressure, orthostatic pulse pressure, orthostatic diastolic blood pressure, orthostatic shock index, orthostatic mean arterial blood pressure, and orthostatic double product.

**Table 5:** Correlation between body mass index and orthostatic cardiovascular responses *P< 0.05

Pearson correlation (r)	Body mass index
Orthostatic pulse rate	0.412
Orthostatic systolic blood pressure	-0.029
Orthostatic diastolic blood pressure	-0.152
Orthostatic pulse pressure	0.134
Orthostatic mean arterial blood pressure	-0.107
Orthostatic shock index	0.364
Orthostatic double product	0.358

Correlation between age and orthostatic cardiovascular responses

According to Table 6, age showed a significant and strong positive correlation with orthostatic systolic blood pressure. Age showed no significant correlation with orthostatic pulse rate, orthostatic pulse pressure, orthostatic diastolic blood pressure, orthostatic shock index, orthostatic mean arterial blood pressure, and orthostatic double product.

**Table 6:** Correlation between age and orthostatic cardiovascular responses *P< 0.05

Pearson correlation (r)	Age
Orthostatic pulse rate	0.0
Orthostatic systolic blood pressure	0.663*
Orthostatic diastolic blood pressure	0.445
Orthostatic pulse pressure	-0.44
Orthostatic mean arterial blood pressure	0.59
Orthostatic shock index	0.364
Orthostatic double product	0.33

## Discussion

The findings of this study suggest that weight and height can be used to predict orthostatic shock index, systolic blood pressure, and pulse pressure in adult healthy female individuals. Like previous studies [3,4,18], 10-min orthostasis was found to cause an increase in systolic blood pressure (SBP), diastolic blood pressure (DBP), pulse rate, mean arterial blood pressure, shock index, and double product compared to their respective baseline readings. The change in these indices of cardiovascular function is widely believed to indicate physiological compensatory mechanisms such as baroreceptor inactivation, activation of the sympathetic nervous system, renin angiotensinogen aldosterone system, and improved secretion of arginine vasopressin and cortisol. These mechanisms are designed to reduce diversion and accumulation of blood in the lower extremities, thus improving blood supply to the brain.

Even though SBP and DBP increased significantly following 10 minutes of orthostasis compared to baseline, the pulse pressure remained unchanged. Peripheral oxygen saturation, an index that measures the percentage of oxygenated hemoglobin [20], was decreased after 10 minutes of standing compared to baseline from 100% to 97%. This orthostasis-induced reduction could be attributed to a decrease in blood volume due to a change in cardiac output. Cardiac output is one of the factors that determine oxygen saturation. Prolonged orthostasis reduces venous return and, thus, cardiac output. This trend has been widely reported in literature [4,21].

The major finding of this study was that weight and height can be used to predict orthostatic cardiovascular responses in young female adult individuals. For instance, body weight showed a strong positive correlation with the orthostatic shock index in healthy individuals. A study by Nelson et al. indicated that obese trauma individuals exhibit a higher risk of early hypovolemic shock due to a reduction in venous return caused by upright posture [25]. As the shock index is a direct function of heart rate, it, therefore, implies that healthy female individuals with high body weight demonstrate high shock likelihood following prolonged orthostasis.

Although body mass index did not correlate with orthostatic cardiovascular responses, the study showed that height was inversely correlated with systolic blood pressure and pulse pressure. A United States study that recruited 12,988 participants had earlier revealed that greater height was associated with lower systolic blood pressure and pulse pressure [26]. In essence, the finding of the present study means that taller female individuals may exhibit lower systolic blood pressure and pulse pressure after prolonged orthostasis.

It is widely known that age is not an anthropometric index. In the study, correlation analysis was done between age and orthostatic cardiovascular response. A strong positive correlation was found between age and orthostatic systolic blood pressure. Systolic blood pressure is known to vary with age [27]. The result suggests that as the age of the female individuals increases, a significant rise in systolic blood pressure following prolonged standing becomes prominent.

Besides being a physical activity, standing improves posture, helps align the vertebral column, and strengthens anti-gravity muscles [8,24]. A study by Ma et al. showed that standing from a sitting position alleviated shoulder and neck pain [16]. Standing also serves as an alternative posture for sitting and lying. Thus, intermittent standing plays a significant role in the control of sedentary behavior, obesity, and type II diabetes mellitus. Standing intervention from the sitting position was shown by Bodker et al. to cause a reduction in triglyceride and insulin resistance [28]. Typically, short and long-term responses to prolonged orthostasis include the inactivation of baroreceptors with attendant sympathetic stimulation, activation of the renin-angiotensin-aldosterone system, and release of cortisol and antidiuretic hormone [1,29,30].

Despite this study providing gainful insight into the clinical significance of orthostatic responses, there were a few limitations. Recruiting a small sample size with only healthy participants in the study may potentially exclude some of the target population with a high risk for low orthostatic response. Hence, the reason we recruited female subjects was due to their lower orthostatic tolerance. Further studies can build on this by having a larger sample size spanning all genders in healthy and diseased populations.

## Conclusions

Clinically, orthostatic cardiovascular responses are highly relevant in conditions such as shock, autonomic dysfunctions, and cardiovascular diseases. Patients with autonomic failure, including those with diabetic autonomic neuropathy or neurodegenerative disorders, exhibit abnormal BP and pulse rate responses to orthostasis, predisposing them to syncope and falls. A persistently elevated shock index may indicate hemodynamic instability, as seen in early-stage shock, where pulse rate increases disproportionately to systolic BP. Similarly, the double product is a valuable indicator of myocardial oxygen demand, which can be crucial in evaluating ischemic heart disease, particularly during stress testing. Abnormal orthostatic responses, influenced by anthropometric variations, can help predict cardiovascular risk and guide interventions for high-risk populations. Beyond disease states, these indices have possible implications for occupational health, particularly in professions requiring prolonged standing or rapid positional changes. Individuals with altered orthostatic cardiovascular responses may be more susceptible to orthostatic intolerance, dizziness, and syncope, affecting job performance and safety. Occupational screening using these indices can help identify at-risk individuals, allowing for tailored preventive measures such as hydration strategies, compression therapy, or physical conditioning programs. Additionally, assessing orthostatic responses in relation to body composition can help refine cardiovascular risk assessments, contributing to personalized medical interventions and improved long-term health outcomes.
